# Transcriptome analysis provides new insights into plants responses under phosphate starvation in association with chilling stress

**DOI:** 10.1186/s12870-021-03381-z

**Published:** 2022-01-11

**Authors:** Xiaoning Gao, Jinsong Dong, Fatemeh Rasouli, Ali Kiani Pouya, Ayesha T. Tahir, Jun Kang

**Affiliations:** 1 School of life sciences, Tianjin University, No.92 Weijin Road, Nankai District, 300072 Tianjin, China; 2 Shanghai Center for Plant Stress Biology and Center of Excellence for Molecular Plant Sciences, Chinese Academy of Sciences, No. 3888 Chenhua Road, 201602 Shanghai, P. R. China; 3 Department of Biosciences, COMSATS University Islamabad, Park road, 45550 Islamabad, Pakistan

**Keywords:** Phosphate starvation, Chilling stress, STOP1, ALMT1, Fe accumulation

## Abstract

**Background:**

Chilling temperature reduces the rate of photosynthesis in plants, which is more pronounced in association with phosphate (Pi) starvation. Previous studies showed that Pi resupply improves recovery of the rate of photosynthesis in plants much better under combination of dual stresses than in non-chilled samples. However, the underlying mechanism remains poorly understood.

**Results:**

In this study, RNA-seq analysis showed the expression level of 41 photosynthetic genes in plant roots increased under phosphate starvation associated with 4 °C (-P 4 °C) compared to -P 23 °C. Moreover, iron uptake increased significantly in the stem cell niche (SCN) of wild type (WT) roots in -P 4 °C. In contrast, lower iron concentrations were found in SCN of *aluminum activated malate transporter 1 (almt1)* and its transcription factor, *sensitive to protein rhizotoxicit*y 1 (*stop1*) mutants under -P 4 °C. The Fe content examined by ICP-MS analysis in -P 4 °C treated *almt1* was 98.5 ng/µg, which was only 17% of that of seedlings grown under -P 23 °C. Average plastid number in *almt1* root cells under -P 4 °C was less than -P 23 °C. Furthermore, *stop1* and *almt1* single mutants both exhibited increased primary root elongation than WT under combined stresses. In addition, dark treatment blocked the root elongation phenotype of *stop1* and *almt1*.

**Conclusions:**

Induction of photosynthetic gene expression and increased iron accumulation in roots is required for plant adjustment to chilling in association with phosphate starvation.

**Supplementary Information:**

The online version contains supplementary material available at 10.1186/s12870-021-03381-z.

## Background

Phosphorus (P) is one of the macronutrient notably essential for the growth of plants. Plants have to evolve strategies such as efficient use of inorganic phosphate (Pi), the major form of P available from soil, to overcome the low Pi availability in many agricultural systems [1]. Photosynthesis inhibition under -P conditions is one of the mechanisms reported to overcome this deficiency. Under -P condition the energy-transducing system is impaired, consequently inhibiting several Calvin cycle enzymatic activities [2, 3]. Deficiency of this macronutrient inhibits triose-P translocation from stroma to cytosol and results in increased production of starch and release of Pi. Thereafter, this Pi is mainly consumed for ATP generation in the light reaction of photosynthesis. However, inhibition of photosynthesis leads to conservation of Pi under its -P condition. Several microarray studies unveiled the down regulation of photosynthetic genes in plants under Pi depletion [4, 5].

Chilling stress is also among one of the abiotic stresses responsible for low productivity and limited crop growth [6]. The efficiency of photosynthetic electron transport in plants is reduced by low temperatures and in combination with high light treatment could even lead to photoinhibition by implication of photosystem I (PSI) instead of PSII [7, 8]. The PS I system is the major storage place of plant iron (Fe), an important micronutrient for various plant functions. Hence, alterations in system functioning can lead to change in homeostasis of Fe as well as oxidation-reduction reactions controlled by iron-sulfur (Fe-S) centers (Fe-Sx, FeSA, and FeSB) in the PS I system [9].

When plants are grown in transparent petri dishes under Pi deficient (-P) condition, primary root elongation will be inhibited as the accumulation of hydroxyl radicals caused by Fe redox cycle in the root apoplast [10]. Plants mostly accumulate high Fe levels in the maturation zone of roots, while in stem cell niche (SCN) and cortex of roots the level is usually very low [5, 11]. The Fe accumulation pattern for plants under Pi deficiency in association with chilling is largely unknown.

In *Arabidopsis thaliana*, several proteins play pivotal roles in Fe accumulation. During Pi starvation, interaction between *LPR* (*Low Phosphate Root*) and *PDR2 (Phosphate Deficiency Response 2*) facilitate cell-specific apoplastic Fe and callose deposition in both the meristem and elongation zone of primary roots [12, 13]. The *STOP1* (*Sensitive to Protein Rhizotoxicity)* transcription factor regulates the expression of the plasma membrane-localized *Aluminum Activated Malate Transporter 1* (*ALMT1*). A previous study showed that phosphate depletion activates STOP1-ALMT1, which rapidly results in inhibition of root cell elongation [14]. The LPR1–PDR2 module interacts with an unknown pathway parallel to STOP1–ALMT1 and inhibits cell division in SCN by a similar mechanism involving Fe accumulation [12, 15]. Implication of STOP1 and ALMT1 under Pi, aluminium and Fe stress is known, their entanglement under a combination of -P and chilling stress has not yet been reported.

In study presented here, we showed that the genes controlling the Fe homeostasis in root SCN are involved in the recovery of photosynthesis rate for -P 4 °C treated plants. Fe accumulation pattern in *stop1,almt1* and *lpr1lpr2* mutants, RNA-seq and qPCR data of photosynthesis related genes and TEM observations for starch granules and chloroplast helped us to unravel the role of STOP1 and ALMT1 under action of dual stress.

## Results

### RNA-seq analyses of the root transcriptome under chilling stress and Pi starvation

**Fig. 1:**
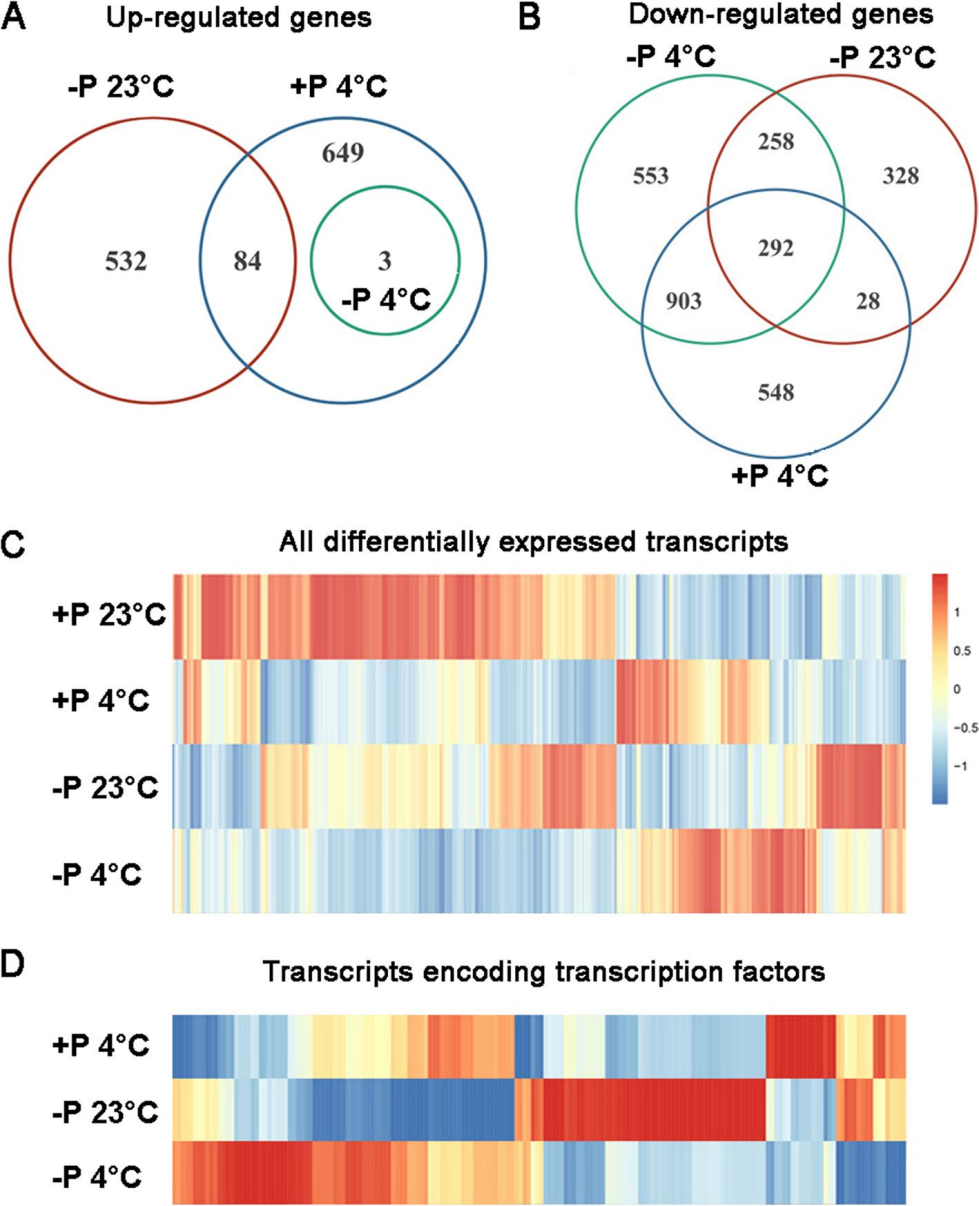
Differentially expressed transcripts in the roots of 7-day-old seedlings grown under 23 °C/ 4 °C in association with +P / -P condition. **A** Venn plot of up regulated genes. **B** Venn plot of down-regulated genes by Pi deficiency and 4 °C. **C** Expression patterns of all transcripts that were differentially expressed in our analysis. **D** Expression patterns of differentially expressed transcription factors

To elucidate the molecular mechanisms under combined action of cold stress and Pi starvation (-P) in plant roots, a comparative RNA-seq analysis of root transcriptome was performed. cDNA libraries were constructed from the roots of 7-day-old WT seedlings using the total RNA, which were divided into four groups according to the growth condition to which they were subjected: +P medium, -P medium, 23 °C, or transferred to 4 °C for 24 h. Using an Illumina HiSeq sequencer, low quality reads as well as those that aligned to either rRNA or tRNA were removed. Total of 247,236,020 reads were selected for additional analysis (Table S1). The raw data of Illumina reads is available at the NCBI Sequence Read Archive browser (http://ncbi.nlm.nih.gov/sra, accession no. PRJNA609588). For each sample, ~95.86% of reads mapped to the *Arabidopsis* TAIR10 reference genome. Of these mapped reads, ~93.57% were aligned to unique genes without ambiguity. RNA expression profiles gross comparisons against +P 23 °C WT roots are shown in Fig. 1 A-D .

First, we compared the transcriptome of the roots of WT seedlings under -P 23 °C and +P 23 °C. In -P 23 °C wild type (WT) seedlings, expression was induced for 616 genes and repressed for 906 genes (Fig. 1 A, B and C). Five *phosphate starvation induced* (*PSI*) marker genes (*At4*, *IPS1*, *PHT1;4*, *SPX1*, and *RNS1*) were found among the induced gene category, as also previously reported by [16]. The fold-change of these five *psi* genes was 4.9, 8.5, 3.2, 4.3, and 5.5, respectively. The induction of these five *psi* genes in Pi-deficient root tips confirmed that the experimental conditions for RNA-seq analysis were appropriate. In -P WT, we also found upregulation of many other known *PSI* genes such as the Pi transporters *Pht1;3, Pht1;7 and Pht1;9*, *purple acid phosphatase 23* (*AtPAP23*) [17, 18]; the Pi-signaling components *SPX3, MIR399B, MIR399C*, and *MIR399D* [19, 20]; copper transport protein family, *LSU2*, an indication of a cross-talk between different nutrients for their uptake and metabolism [21, 22]. Enhanced expression of *BCAT4, MAM1, IPMI1* and *IPMI2* [23–25] suggested elevated biosynthesis of glucosinolates and branched-chain amino acids (Table S2).

In the category of down-regulated genes mainly two functional groups were enriched in -P 23 °C seedlings. One group was dominated by transcription factors (total number: 58) whereas a second group was comprised of photosynthesis-related (PR) genes (total number: 75) (Fig. 1D, Table S3). In the second group we found 18 genes encoding subunits of photosystem I, 17 chlorophyll-binding proteins, 13 genes encoding enzymes involved in the Calvin cycle and 27 genes encoding subunits of PS II (Table S4). None of these photosynthetic genes were up-regulated in -P 23 °C roots, and the expression level of ~70% of these genes was two times higher in +P 23 °C.

The transcriptome of +P 4 °C was then compared to +P 23 °C. In +P 4 °C, 736 genes were upregulated while 1771 genes were downregulated (Fig. 1 A, B and C, Table S5). 92 of 220 transcription factors were up-regulated and 128 were down-regulated (Fig. 1D and Table S6). The largest increase in induction among transcription factors (4.9-fold increase) in +P 4 °C was found for *PRR9* (At2g46790). *PRR7* (AT5G02810) and *PRR9* are partially redundant essential components of a temperature-sensitive circadian system [26]. *CCA1* (At2g46830) encodes a transcriptional repressor that performs overlapping functions with *LHY* (At1g01060) in a regulatory feedback loop that is closely associated with the circadian oscillator of *Arabidopsis*. *CCA1* and *LHY* exerted a positive effect on *PRR9* [27]. Photosynthesis-related genes were mostly down-regulated (52 out of 54), including 9 genes encoding chlorophyll-binding proteins, 15 genes encoding enzymes involved in the Calvin cycle, 13 genes encoding subunits of photosystem I, 17 genes encoding subunits of photosystem II (Table S7).

Finally, the transcriptomes of seedlings grown on -P 4 °C and -P 23 °C media were compared to determine the effects of chilling stress on gene expression profiles in plants under phosphate starvation. 996 genes were up-regulated in -P 4 °C whereas 1676 genes were down-regulated (Fig. 1 A, B and C, Table S8). Genes that belonged to transcription factors and photosynthetic regulation were the most enriched categories. Among the transcription factors, expression of 72 genes was increased and that of 65 genes was decreased in -P 4 °C relative to -P 23 °C. The largest increase in induction among transcription factors (7.8-fold increase) was found for *CBF3*, which encodes a member of the DREB subfamily A-1 of ERF/AP2 transcription factor family [28] (Table S9). Activation of CBF3 triggers feedback on temperature perception by suppressing the premature cold-acclimation at standard temperature. Three zinc finger (B-box type) family proteins (At4g27310, At5g54470, and At3g21890) [29] were also expressed at high level, with inductions of 4.8, 4.2, and 4.2 fold, respectively. Among 48 differentially expressed photosynthesis-related genes, expression of 41 photosynthetic genes was elevated in -P 4 °C compared to -P 23 °C. Highest fold changes among genes involved in photosynthesis were for three *ribulose bisphosphate carboxylase small chains 2B, 3B*, and *1B* [30] with expression levels of 11.7, 8.7, and 6.4, respectively. Elevated expression of four ferritins *(HY2, ATFRO5, FD2*, and *GLU1*) [31–33], indicated that the Fe homeostasis in roots might be enhanced after 4 °C treatment under -P stress. Several important components earlier reported to be involved in the photosystem including *AGT1, PPL1, CAB3, PIF1, GAPA-2*, and *PSAH2* [32, 34–38], were also highly induced (Table S10), indicating that the ability for photosynthesis has been enhanced in plants at 4 °C compared with 23 °C under -P stress.

## Verification of photosynthesis-related and marker gene mRNA expression level

To confirm the mRNA expression of the marker genes in response to Pi starvation and cold stress in roots, qPCR was performed using roots of the 7-day-old seedlings. Eight photosynthesis-related or marker genes were analyzed for their expression in WT grown under different Pi and temperature conditions. These eight genes encode: *AGT1*, a peroxisomal photorespiratory enzyme that catalyzes transamination reactions with multiple substrates and which is involved in photorespiration; *RBCS1B*, a member of the rubisco small subunit (RBCS) multigene family; *IPS1*, a non-coding transcript; *KIN1*, an anti-freeze protein ; *ERF109*, a member of the ethylene response factor (ERF) subfamily B-3 of the ERF/AP2 transcription factor family; *MAPKKK19*, a member of the MEKK subfamily; *PIN5*, a functional auxin transporter that is required for auxin-mediated development. *PIN5* does not have a direct role in cell-to-cell transport but regulates intracellular auxin homeostasis and metabolism; and *COR15A*, whose constitutive expression increases freezing tolerance in protoplasts in vitro and chloroplasts in vivo. As expected, the expressions of *IPS1*, *KIN1*, *MAPKKK19*, *PIN5* and *COR15A* were enhanced by Pi starvation or cold stress (Figure S1); interestingly, the induction level of *AGT1* and *RBCS1B* was much higher in -P 4 °C than in -P 23 °C and +P 4 °C, and even 3-4 times higher than that in +P 23 °C.

### ***stop1*** and ***almt1*** accumulated less Fe^3+^ than WT in roots under chilling in association with Pi starvation stress

**Fig. 2:**
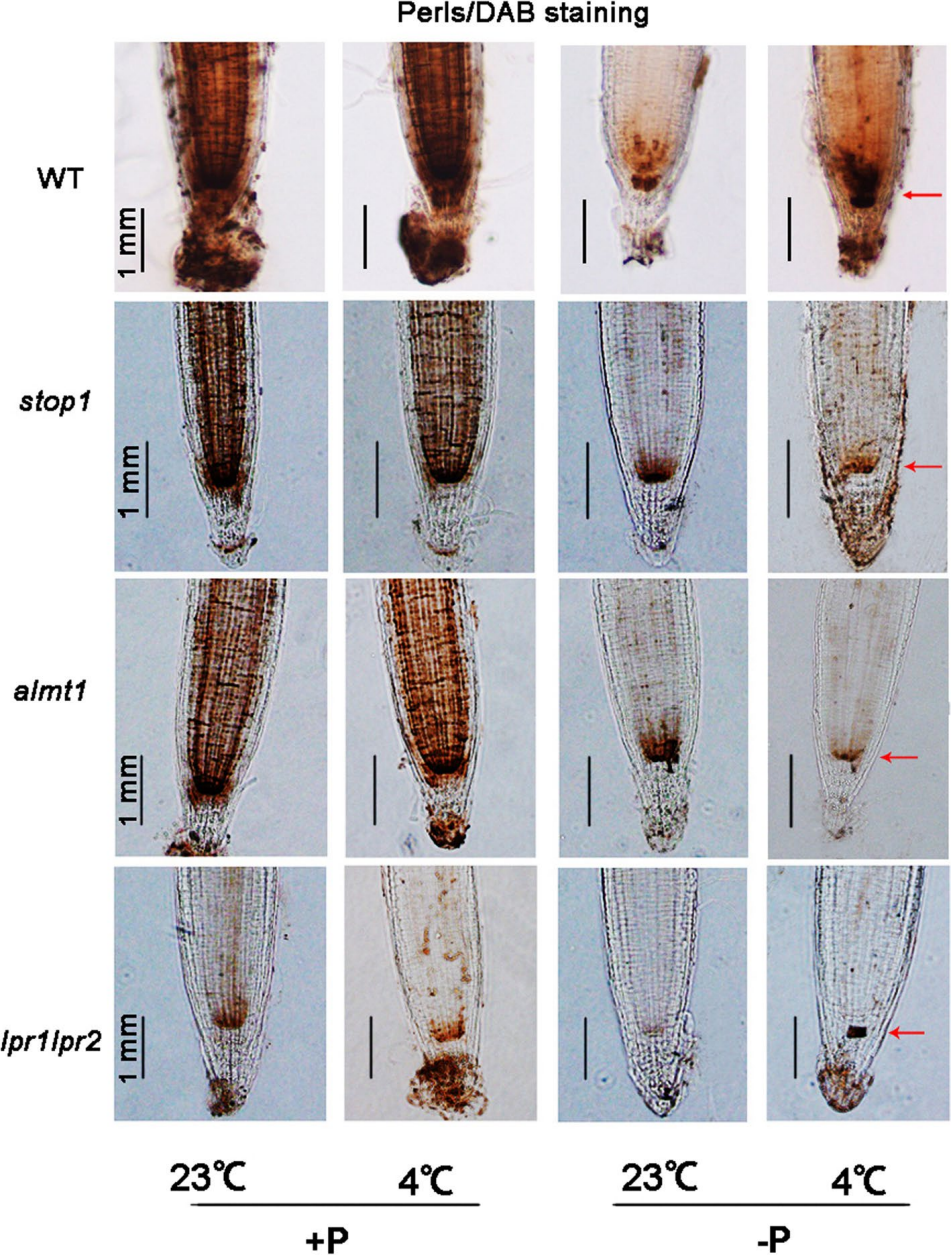
Histochemical staining by Perls/DAB for determination of Fe accumulation patterns on +P 23 °C, -P 23 °C, +P 4 °C and -P 4 °C media. Root samples of 7-day-old seedlings were used

Previous studies showed that iron–sulfur (Fe-S) compounds (ferritin) played a significant role in the PS I process. These proteins are pivotal centers for Fe storage in plants. To identify whether the increased expression level of ferritins in -P 4 °C plants was attributable to Fe absorption, the Fe accumulation pattern was identified in *Arabidopsis* roots under four treatment conditions. Seeds were sown on +P and -P media at 4 and 23 °C. Perls/DAB (diaminobenzidine) staining method which stains Fe^2+^ as well as Fe^3+^. Clearly, the WT seedlings grown on +P 23 °C and +P 4 °C showed dark brown Fe staining (Fig. 2). There was no significant difference between the two treatments. For seedlings grown on -P 23 °C, Fe^3+^ accumulation in the SCN, cortex, and root apex was strongly decreased, instead, darker brown staining in -P 4 °C treated WT roots was much sharper compared to that of -P 23 °C plants (Fig. 2). This result demonstrated an increased Fe accumulation in root tips under -P 4 °C compared with -P 23 °C, indicating the impact of chilling stress on Fe homeostasis in root SCN.

Seeds of *stop1, almt1* and *lpr1lpr2* were also sown on both +P and -P media as they have been reported to paly pivotal role in Fe homeostasis. Results showed that, Fe accumulation in roots of all mutants showed almost no difference between 23 °C and 4 °C under +P condition. With -P treatment, Fe deposition in the quiescent center (QC) of *lpr1lpr2* in at 4 °C increased and was notably stronger in two or three cells than at 23 °C (Fig. 2). However, in *stop1* and *almt1* mutants, the Fe distribution in SCN was lower in -P 4 °C compared to -P 23 °C.

**Fig. 3:**
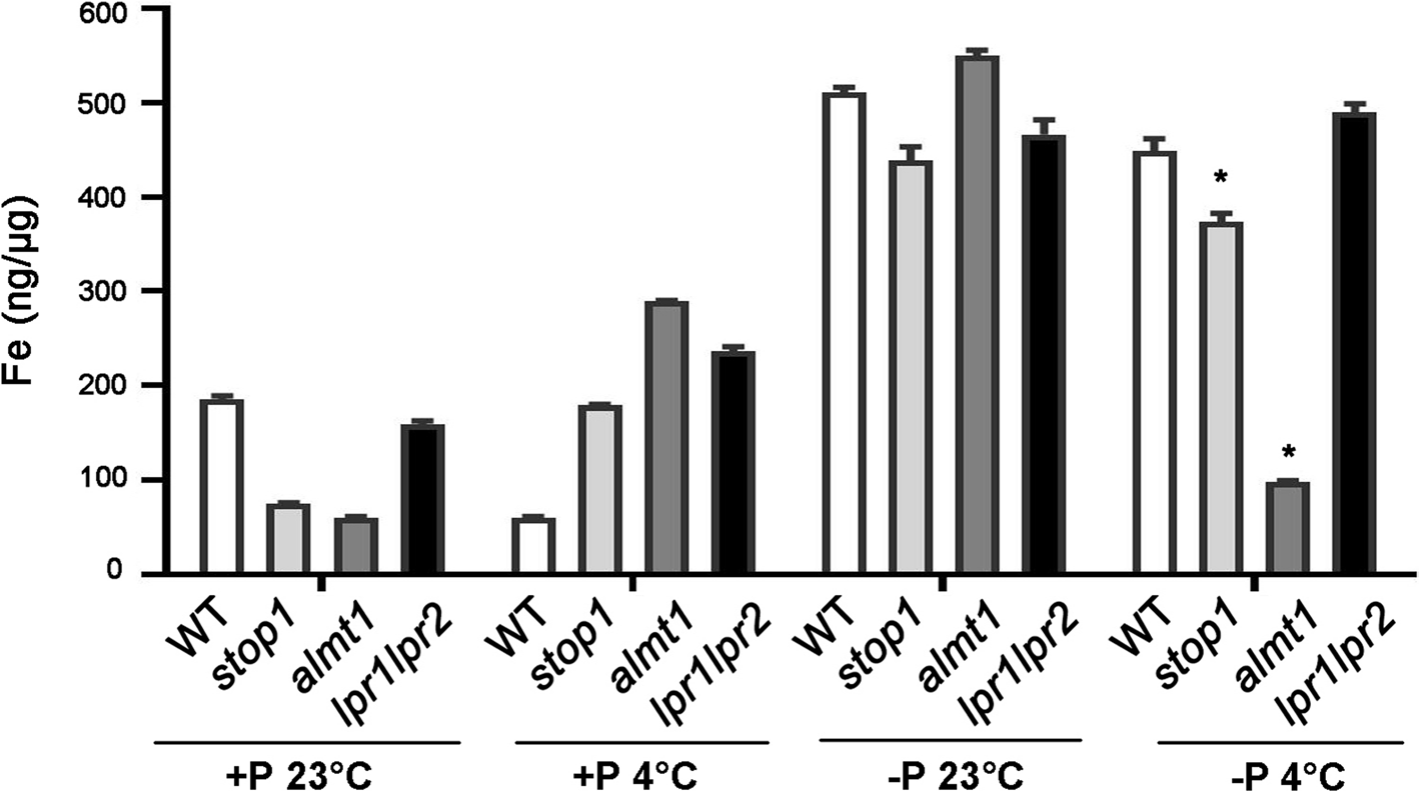
Quantification of Fe content in WT, *stop1, almt1* and *lpr1lpr2* seedlings grown on +P 23 °C, -P 23 °C, +P 4 °C and -P 4 °C media. 10-day-old seedlings were used to take the roots for assay. The values are means ± SD of three biological replicates. *(*t*-test, P < 0.05)

Quantitative analysis indicated that the total Fe content was about three times higher in -P 23 °C seedlings of WT compared to that for +P 23 °C (Fig. 3). -P 4 °C treated WT and *lpr1lpr2* Fe contents were 448.5 ng/µg and 489.5 ng/µg respectively. However, the Fe content in -P 4 °C treated *almt1* was only 98.5 ng/µg. In -P 4 °C treated *stop1*, Fe content was 373.5 ng/µg (Fig. 3). These results suggested that the enhanced Fe accumulation in plant root tips under -P 4 °C might be caused by ALMT1 and STOP1.

### ***stop1*** and ***almt1*** mutations lead to suppressed PR phenotypes under -P 4 °C

**Fig. 4:**
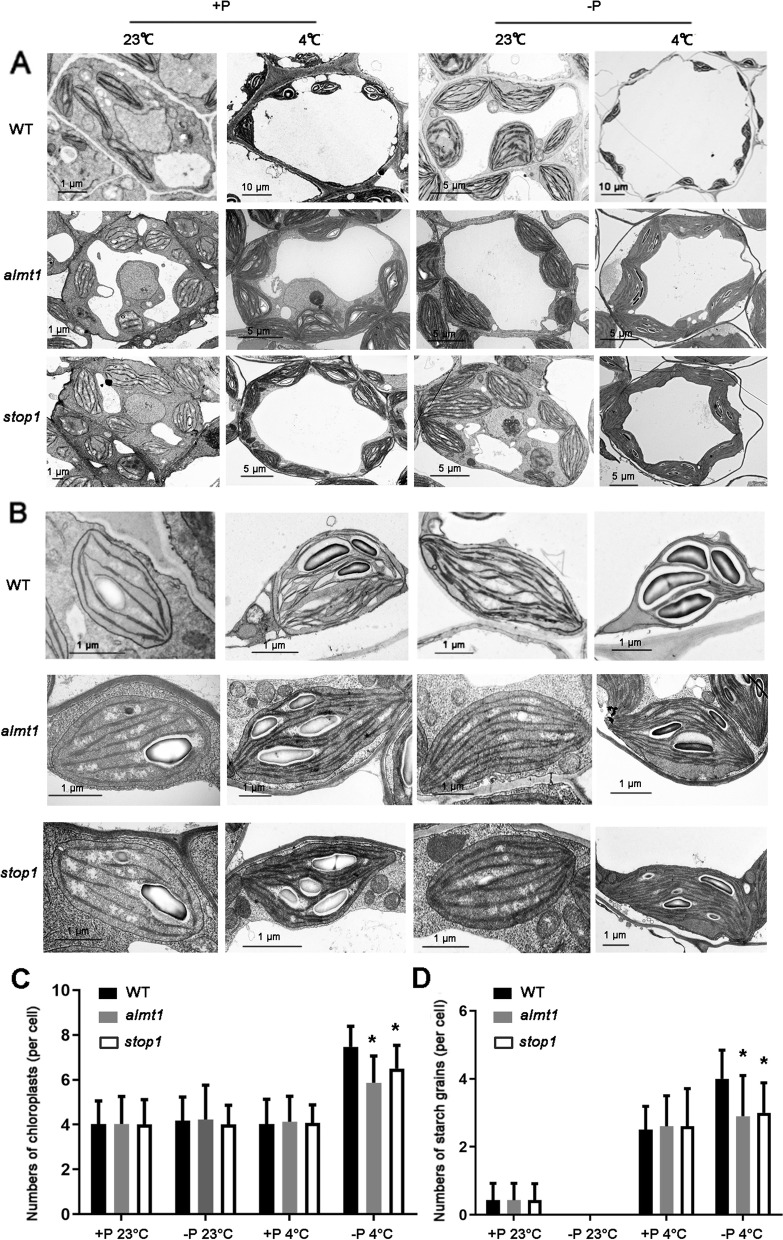
Shoot apical meristem chloroplast and starch grain contents of WT, *almt1* and *stop1* seedlings grown on +P 23 °C, -P 23 °C, +P 4 °C and or -P 4 °C conditions. Samples were analyzed at 10 DAG, by transmission electron microscopy (TEM). **A** TEM micrographs of chloroplast in one cell; **B** the accumulation of starch grains in one chloroplast; **C** number of chloroplast per cell and starch grains per chloroplast. In **(B)** and (**C**), values are means of 30 cells ± SD. Bars=1 μm

To ascertain whether the relatively increased expression of photosynthetic genes actually impact the photosynthesis-related phenotypes in plants, the chloroplast development in shoot apical meristems was examined via transmission electron microscopy (TEM). The results are shown in Fig. 4. There were some similar phenotypes among WT, *almt1* and *stop1*. For instance, the cell wall was very firm in all types of plants under +P 23 °C treatment, and every cell contained ~4 chloroplasts with well-developed grana stacks, with the average number of starch granules in each chloroplast was approximately 0.4. Under +P 4 °C treatment, cell walls looked looser than those under +P 23 °C treatment. An average of four chloroplasts were found per cell with less well developing grana stacks than in the +P 23 °C treatment. The average number of starch granules increased to ~2.5 per chloroplast. With the -P 23 °C treatment we obtained an average of 4.2 chloroplasts per cell with well-developed grana stacks, though lacking the starch granules.

In addition to the number of chloroplasts, the accumulation pattern of starch granules in *almt1* and *stop1* under -P 4 °C were much different from those in WT. The average number of chloroplasts in WT was 7.2 per cell, and all contained well developed starch granules with an average number of 4.0 per chloroplast (Fig. 4). However, there were only 5.9 and 6.2 chloroplasts per cell in *almt1* and *stop1* with poorly developed starch granules, 2.9 and 3.0 respectively. In addition, the cell wall of WT was looser than those of *almt1* and *stop1*. The chloroplasts in the two mutants were arranged more tightly compared to their arrangement in WT (Fig. 4).

**Fig. 5:**
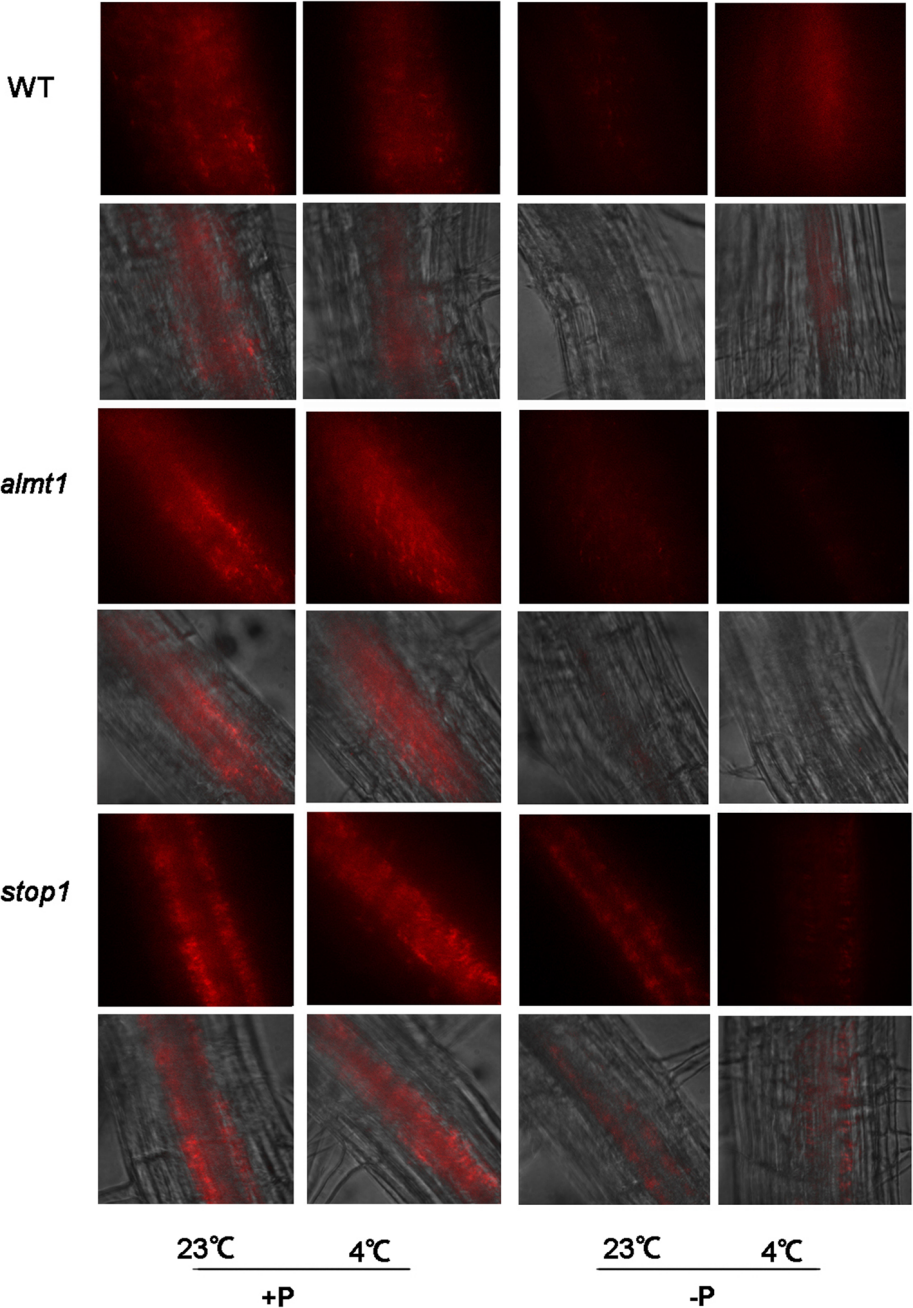
Chlorophyll accumulation patterns in *almt1, stop1* and WT. Primary roots were excised from 10-day-old seedlings and were photographed by confocal laser microscopy. In each panel, chlorophyll auto-fluorescence (red) is depicted in the top row whereas the bright field images are presented in the bottom row

Patterns of chlorophyll auto-florescence of 10-day-old seedling roots were also analyzed. Compared to  WT under -P 4 °C, the signal intensity in *almt1* and *stop1* mutants could hardly be detected (Fig. 5), which indicates the poor root plastid development under dual stress. These findings indicated that mutation of *almt1* and *stop1* suppressed the photosynthetic reaction induced by -P 4 °C.

### ***stop1*** and ***almt1*** showed promoted primary root elongation under phosphate starvation associated with chilling stresses

**Fig. 6:**
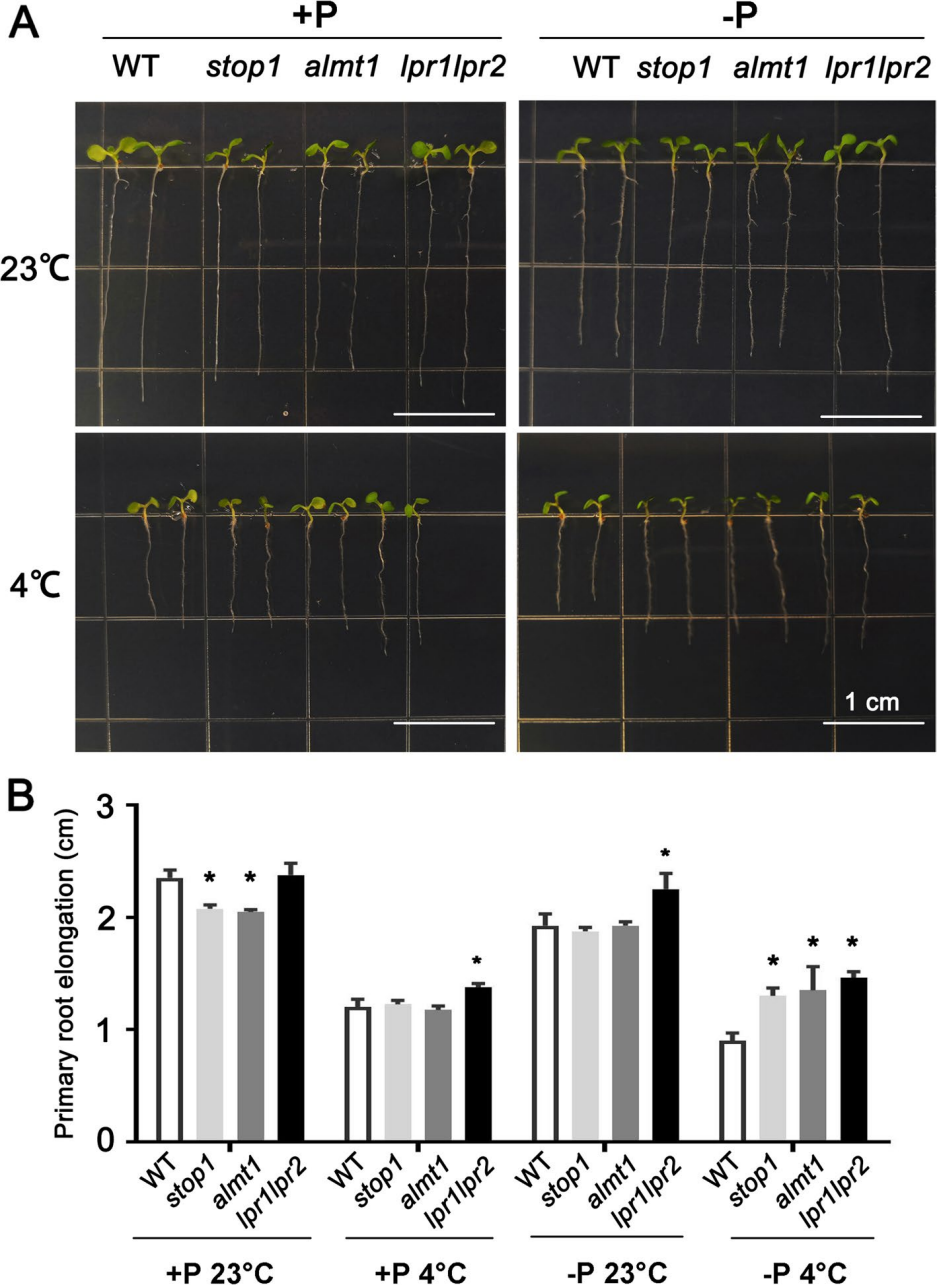
Primary root elongation under +P 23 °C, -P 23 °C, +P 4 °C and -P 4 °C conditions. (**A**) Phenotype of 7-day-old WT, *stop1, almt1* and *lpr1lpr2* seedlings; (**B**) Quantification of primary root elongation of plants. The values are means ± SD of ten biological replicates. *(*t*-test, P < 0.05)

Over-accumulation of Fe always leads to inhibition of primary root elongation. In contrast, lower concentration of Fe deposit leads to insensitive root elongation phenotype under phosphate starvation stress [12]. To detect the primary root elongation condition under combined stresses, the WT, *stop1*, *almt1* and *lpr1lpr2* seeds were planted in +P and -P media (both at two temperatures 4 and 23 °C). Results showed that primary root elongation was inhibited in *stop1* and *almt1* under +P associated with 23 °C (+P 23 °C) treatment. Under -P 23 °C and +P 4 °C, the difference of primary root elongation among WT and *stop1* and *almt1 *was not obvious (Fig. 6 A). However, the length of primary root was longer in *stop1* and *almt1* compared with WT under -P 4 °C condition (Fig. 6 A). Quantitative data revealed that the difference of primary root length among WT, *stop1* and *almt1* was significant (Fig. 6B).

### Dark treatment blocked the primary root elongation phenotype of ***almt1*** and ***stop1*** under -P 4 °C

**Fig. 7:**
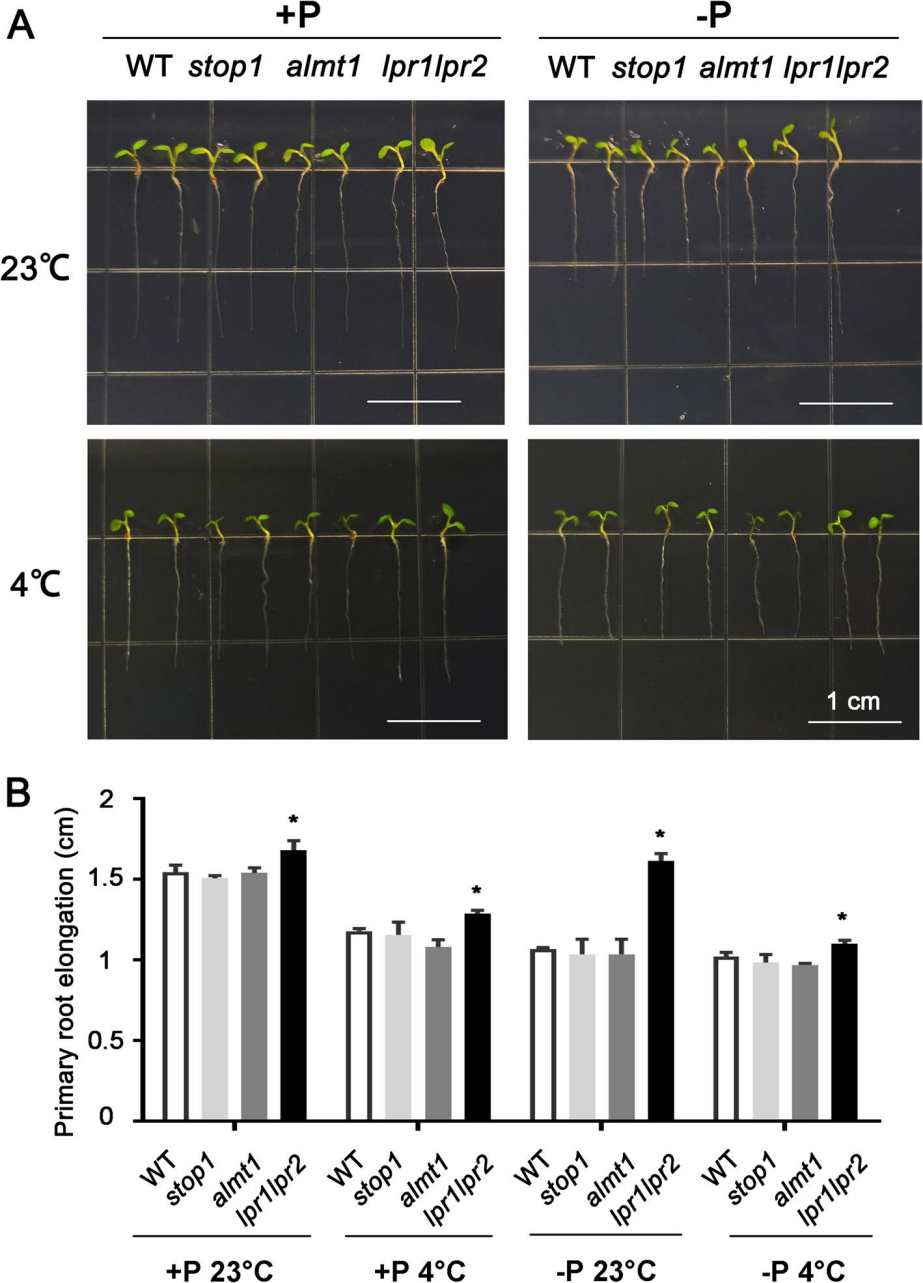
Primary root elongation with dark treatment under +P 23°C, -P 23°C, +P 4°C and -P 4°C conditions. (**A**) Phenotype of 7-day-old WT, *stop1, almt1 *and *lpr1lpr2* seedlings; (**B**) Quantification of primary root elongation of plants. The values are means ± SD of ten biological replicates. *(*t*-test, P < 0.05)

To further investigate the primary root elongation when photosynthesis was inhibited, the dark treatment with combined stresses was conducted. The WT, *stop1*, *almt1* and *lpr1lpr2* seeds were planted in +P and -P media and cultured under 4 and 23 °C, respectively (Fig. 7 A). It was easy to find that there was no obvious difference in primary root elongation of the WT, *stop1* and *almt1* under four conditions. Quantitative analysis of primary root length further revealed that the difference among WT, *stop1*, *almt1* was not significant (Fig. 7B). Clearly, dark treatment blocked the function of *almt1* and *stop1* in Fe homeostasis under -P 4 °C.

## Discussion

Reduced photosynthesis rate in plants under Pi starvation or cold stress has been observed more than 20 years ago [7, 16]. When low-Pi plants are resupplied with Pi, the rate of photosynthesis and stomatal conductance in chilled tomato plants recovered much better (approximately twice as well) than in non-chilled samples. Although plants over-accumulate Fe under Pi starvation stress, little Fe was found accumulated in the SCN and cortex of roots [11]. This study provided first evidence that Fe storage was enhanced in SCN of WT, especially in QC, under -P associated with cold stress. Consequently, increased Fe accumulation mediated by ALMT1 and STOP1 led to the inhibition of primary root elongation and aided for plant adjustment to combinations of chilling stress and phosphate deficiency.

Fe is a pivotal macronutrient for PS I process, as it is important for the synthesis of Fe-S proteins working in PS I reaction center [9]. Shortage of Fe would lead to the decreased efficiency of light reaction processes, which results in the inhibition of ATP synthesis, reducing CO_2_ immobilization and reduced starch grain accumulation during dark reaction [39]. On the other hand, over-accumulation of Fe in roots leads to the inhibited primary root elongation as the high level of reactive oxygen species (ROS) result in an increased callose deposition in cell walls and plasmodesmata [11]. In *almt1* and *stop1* mutants, the Perls/DAB staining of roots under -P 4 °C was much lighter than for those under -P 23 °C (Fig. 3). Clearly, mutation of *almt1* and *stop1* inhibited the Fe accumulation for plant grown under -P 4 °C. However, there was still some Fe accumulation in SCN of *almt1* and *stop1*. Two reasons might be the cause of this result: one is the 4 °C treatment for 24 h was not long enough for the remaining Fe consumption in *almt1* and *stop1*; the other is that some other Fe translocation proteins also involved in basic Fe absorption during -P 4 °C treatment, e.g. LPR1 and LPR2. Fe transporters involved in the maintenance of Fe homeostasis under -P 4 °C treatment needs further experimental validation and verification.

The RNA-Seq analysis was also used to understand the molecular basis of cross talk between Fe absorption, -P and chilling stress. We particularly focused on the -P 4 °C vs. -P 23 °C group. The largest increase in induction among transcription factors in -P 4 °C was 7.8-fold (*CBF3*). The expression level of the same subfamily member, *CBF2*, was also induced. Recently *CBF* (C-repeat binding factor) pathway has been reported to play key role in plant cold acclimation, a process to increase freezing tolerance to low but non-freezing temperatures in certain plants [40]. This suggests that phosphate deficiency increases the tolerance against chilling stress (4 °C) to a certain extent. Combined with the increased Fe content in root SCN, we suggest that induced freezing tolerance might be caused by the increased ratio of photosynthesis under -P 4 °C. Furthermore, three zinc finger (B-box type) family proteins *BBX28, BBX29*, and *BBX31* were also induced. *BBX28* negatively regulates the photomorphogenic development, by interfering the binding of the transcription factor *HY5* to its target gene promoters. The function of *BBX29* and *BBX31* was not clear before. Our results provided evidence that *BBX29* and *BBX31* might be involved in the same pathway along with *BBX28,* for in the inhibition of photomorphogenic development under -P 4 °C [41]. As photomorphogenic development requires a large amount of Fe and Pi to synthesize new proteins and nucleic acids, inhibition of this process was considered to be an active protection regulation for current survival.

In the -P 4 °C vs. -P 23 °C analysis, genes of Rubisco small subunit multigene family (*RBCS2B, RBCS3B, and RBCS1B*) were the most induced genes among the photosynthesis related genes (Table S10). Their function is to produce a sufficient Rubisco content to maintain the photosynthetic capacity of leaves [30]. It is likely that these enhanced photosynthetic genes and the inhibited photomorphogenic development reached a dynamic balance for plant tolerance to dual-stress treatments. Moreover, four ferritin expression levels *HY2* [42], *ATFRO5* [31], *FD2* [33] and *GLU1* [43] were up-regulated, suggesting that Fe homeostasis in roots has been enhanced after -P 4 °C treatment.

Low temperatures disrupt plant cell walls and loosen cell wall structure compared to control, especially in association with -P treatment (Fig. 4). However, the number of chloroplasts in shoot apical meristems was higher in -P 4 °C than in +P 23 °C, -P 23 °C, and +P 4 °C in WT, *almt1* and *stop1*, and was mirrored in the accumulation of starch granules. It is considered that lack of Fe inhibits the efficiency of PS I and low Pi supply limits the ATP synthesis, followed by the decrease in dark reaction ratio.

Dark treatment brings the inhibition of photosynthesis. And it was considered to further suppress the Fe deposition in plant roots. This work provides evidence that the differences in primary root elongation among WT, *stop1* and *almt1* was conducted by light (Fig. 7). Light treatment promotes the photosynthesis process, which leads to Fe accumulation, and finally caused the primary root inhibition. Though most plant roots are under the ground, yet, for aquatic plants studies with light subjected roots are of great importance.

## Conclusions

The findings of this study provide some new insights to understand the essential roles of ALMT1 and STOP1 in regulating primary root elongation by promoting iron accumulation in root stem cell niche to maintain photosynthesis during -P associated with cold stress. More work needs to be carried out to unravel the complicated molecular network involved in regulation of Fe homeostasis and photosynthesis under combined action of dual stress.

## Methods

### Plant materials and growth media

All the *Arabidopsis* material used in this study is of the Columbia-0 background. The T-DNA insertion lines for *almt1* (SALK_009629C), *stop1* (SALK_114108), *lpr1-1*(SALK_016297) and *lpr2-1*(SALK_091930) were obtained from the Arabidopsis Biological Resource Center (https://abrc.osu.edu/). The *lpr1-1lpr2-1* double mutants were generated by crossing *lpr1-1* to *lpr2-1*. Homozygous lines were verified by PCR using specific LP and RP primers: stop1 (SALK_114108) LP TTCATTGGTGAGAACGACTCC, stop1 (SALK_114108) RP ATCTTCTTGTTGGTCGTGGTG, almt1 (SALK_009629C) LP GAAACACTGGTGATGTCGAT, almt1 (SALK_009629C) RP GTGTTGATTATATGATACGA, lpr1-1 (SALK_016297) LP CTCATCGCCAGTAGGTAGCTG, lpr1-1 (SALK_016297) RP ACTCATGGGTGTGAACCAAAG, lpr2-1 (SALK_091930) LP CATAGCCTGGCTCTTGAGTTG, lpr2-1 (SALK_091930) RP GTCATAGCTCAGTCGAATCGC, LBb 1.3 (T-DNA primer for SALK lines) BP ATTTTGCCGATTTCGGAAC.

The medium +P was half-strength MS medium, which has agar 1.2% (w/v) and sucrose 1% (w/v). KH_2_PO_4_ used for +P medium was replaced with K_2_SO_4_ for preparation of -P medium. Seeds were sterilized, thoroughly washed in sterile-distilled water to remove bleach and stratified at 4 °C for 48 h. For germination and further growth, seeds were shifted to media plates and kept in a growth room at 23 °C for 16 h/8 h day night light conditions. After 6 days, half of the seedlings were kept at 4 °C in a refrigerator for +P and -P 4 °C treatment, with the same light conditions as in the growth room. The light intensity of both growth room and refrigerator was 100 µmol m^−2^ s^−1^. Samples were collected to perform further analyses.

For the dark treatment, seedlings were first kept at 23 °C in +P or -P media under the proper light intensity (100 µmol m^−2^ s^−1^) for three days. After normal and consistent germination, seedlings were treated with 23 °C, 4 °C, dark and light condition for another four days, respectively.

### RNA-seq library preparation

Total RNA was extracted using roots of 7-day-old seedlings by Trizol method (Invitrogen Lige Technologies), with two biological replicates (~100 pooled roots/replicate). After quality check, the preparation of RNA-Seq libraries was performed according to [16].

### Analysis of RNA-seq data

Reads with primer/adaptor contamination, PHRED quality score lower than 20, and those with ambiguous bases were removed. Filtered reads were mapped with TopHat2 (version 2.0.8) against *Arabidopsis* TAIR10 reference genome. Two mismatches were allowed for analyses with minimum intron length of 60nt and that of maximum was 6000 nt. Reads that aligned to rRNA or tRNA were removed. Cuffdiff2 mapping outputs were used to calculate expression counts of gene locus. Normalization was performed against RPKM by edgeR package [44]. Expression was considered significant with a cutoff value of 0.5 RPKM [45]. EdgeR was also used to determine differential expression, where p-value ≤ 0.01 and fold change >2 were criteria of significant change. Differentially expressed genes were clustered using all the detected transcripts or individual functional groups. After log_2_-transformation of all the ratio values, Cluster and Treeview programs were used to perform centroid linkage hierarchical clustering [46]. The raw data has been submitted, the accession number is PRJNA609588.

### Quantitative real-Time PCR analysis

For quantitative real-time PCR (qPCR) analysis, total RNA was extracted from 7 days old seedlings using TIANGEN RNAprep pure plant kit (Tiangen Co., Beijing) according to previously reported method of [16]. The primer sequences used for detection of the mRNA expression of phosphate starvation induced and photosynthesis related genes are listed in Table S11.

### Fe histochemical staining assay

Perls staining method was used to detect Fe in roots, mainly stains labile (non-heme) Fe^3+^, as described by [47]. Briefly, roots from 7-day-old seedlings were vacuum infiltrated with Perls staining solution having equal volumes of 4% (v/v) HCl and 4% (w/v) potassium ferrocyanide. After 15 min of infiltration, the samples were kept in the same solution at room temperature for another 30 min. Thereafter samples were rinsed with ultrapure water to stop the reaction. HCG clearing solution with1 g/ml chloral hydrate in 15% glycerol was used and finally cleared samples were examined with a DIC microscope. DAB intensification was performed as described by [11].

### Fe content quantification in roots

Root tissues were used to detect Fe content by bathophenanthrolinedisulfonic acid colorimetric assay as described by [48].

### Transmission electron microscopy of shoot apical meristems

For transmission electron microscopy we used shoot apical meristems from 10-day-old seedlings. Seeds were kept in growth room at 23 °C for 16/8 h day night light conditions. Six days later, half of the seedlings were kept at 4 °C refrigerator with +P or -P 4 °C treatments, for four days. The second half of seedlings were kept at +P or -P 235 °C. Samples were fixed in 2.5% glutaraldehyde for 24 h according to [16]. Stained shoot apical meristems were analyzed using the transmission electron microscope (H-7650B, Hitachi).

### Detection of Chlorophyll autofluorescence

Chlorophyll autofluoescence was detected in roots of 10-day-old seedlings using confocal laser scanning microscope (LSM710; Carl Zeiss) at excitation wave length of 488 nm.

### Accession numbers

Sequence data from this article can be found in the TAIR database under the following accession numbers: ALMT1 (At1g08430), STOP1 (At1g34370), LPR1 (AT1G23010), LPR2 (AT1G71040), IPS1 (At3g09922), KIN1 (At5g15960), ERF109 (At4g34410), MAPKKK19 (At5g67080), PIN5 (At5g16530), COR15A (At2g42540), CBF2 (At4g25470), CBF3 (At4g25480), BBX28 (AT4G27310), BBX29 (At5g54470), BBX31 (At3g21890), RBCS (At5g38430), AGT1 (At2g13360), GOX1 (At3g14420), PSAH2 (At1g52230), HY2 (AT3G09150), FED A (At1g60950).

## Supplementary Information

**Additional file 1: Figure S1.** Relative phosphate starvation and chilling stressinduced gene expression (as determined by qPCR) in 7-day-old seedlings of WTgrown on +P and -P media under 23°C and 4°C. Values for each gene are relative to theexpression in WT on +P 23°C, set at 1.0. Values are the means ± SD of three biological replicates. An asterisk indicates a significant difference(p<0.05, *t*-test) from the WT.

**Additional file 2: Table S1.** Table of all mapped RNA reads.

**Additional file 3: Table S2.** Differentially expressed transcripts in -P 23°C compared to +P 23°C plants.

**Additional file 4: Table S3.** Differentially expressed transcription factors in -P 23°C compared to +P 23°C plants.

**Additional file 5: Table S4.** Differentially expressed photosynthesis related genesin -P 23°C compared to +P 23°C plants.

**Additional file 6: Table S5.** Differentially expressed transcripts in +P 4°C compared to +P 23°C plants.

**Additional file 7: Table S6.** Differentially expressed transcription factors in +P 4°Ccompared to +P 23°C plants.

**Additional file 8: Table S7.** Differentially expressed photosynthesis related genesin +P 4°C compared to +P 23°C plants.

**Additional file 9: Table S8.** Differentially expressed transcripts in -P 4°C comparedto -P 23°C plants.

**Additional file 10: Table S9.** Differentially expressed transcription factors in -P 4°Ccompared to -P 23°C plants.

**Additional file 11: Table S10.** Differentially expressed photosynthesis related genesin -P 4°C compared to -P 23°C plants.

**Additional file 12: Table S11.** Sequences of the primers used for quantitative PCR.

## Data Availability

The materials of this study were provided by School of Life Sciences, Tianjin University. Correspondence and requests for materials should be addressed to Jun Kang (jun.kang@tju.edu.cn).

## Abbreviations

Pi: phosphate
-P: phosphate starvation
PS: photosystem
QC: quiescent center
SCN: stem cell niche
*almt1*: *aluminum activated malate transporter 1*
*stop1*: *sensitive to protein rhizotoxicit*y 1
ROS: reactive oxygen species
qPCR: quantitative real-time PCR
