# Supplementary figures and images for: Transcriptome analysis provides new insights into plants responses under phosphate starvation in association with chilling stress
Source: BMC Plant Biol. 2022 Jan 11;22:26. doi: 10.1186/s12870-021-03381-z (PMC8751124; doi:10.1186/s12870-021-03381-z)

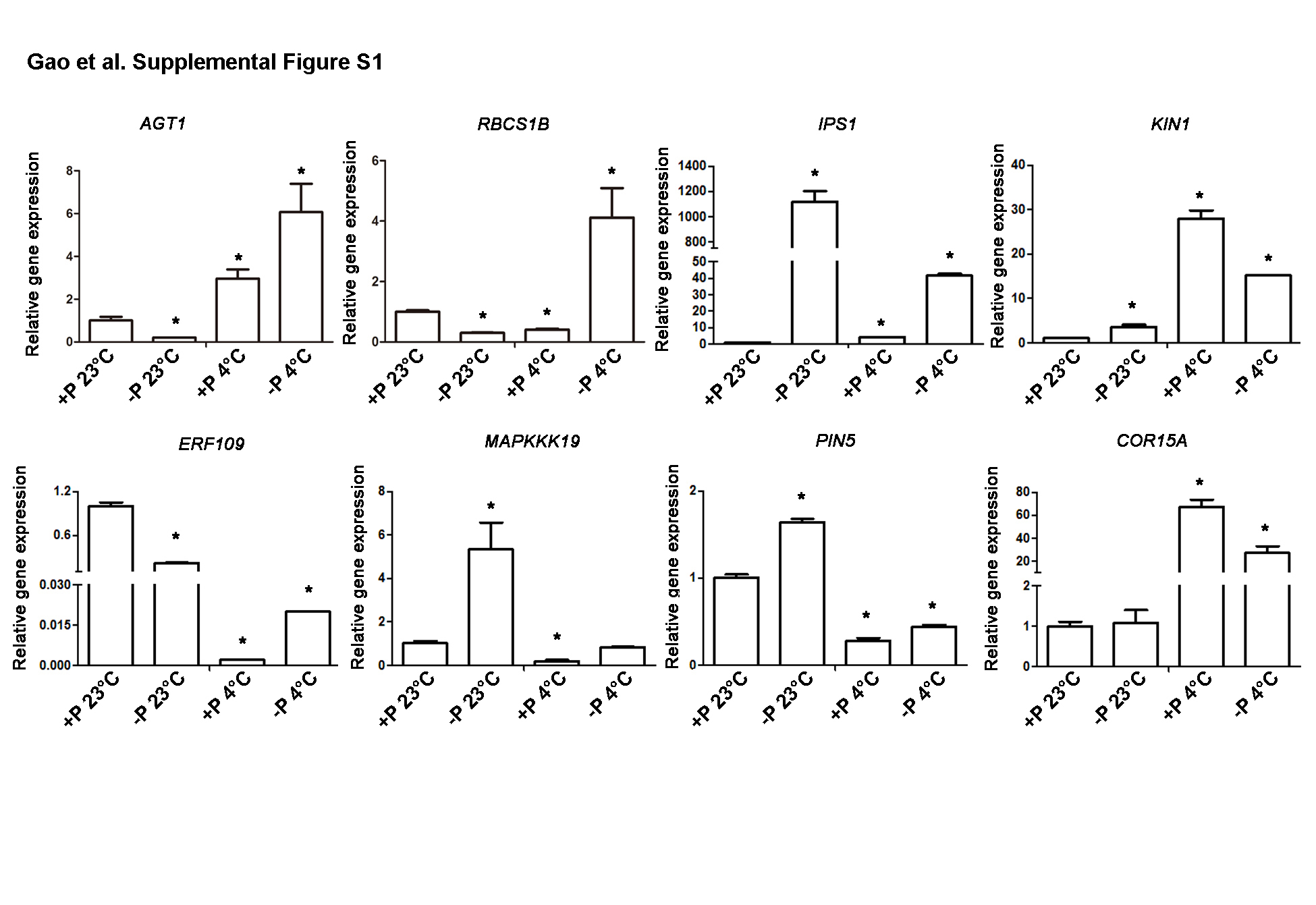

Supplement: Supplementary file 1 — Additional file 1: Figure S1. Relative phosphate starvation and chilling stressinduced gene expression (as determined by qPCR) in 7-day-old seedlings of WTgrown on +P and -P media under 23°C and 4°C. Values for each gene are relative to theexpression in WT on +P 23°C, set at 1.0. Values are the means ± SD of three biological replicates. An asterisk indicates a significant difference(p<0.05, t-test) from the WT. [file 12870_2021_3381_MOESM1_ESM.jpg]
